# Real-time dense small object detection algorithm based on multi-modal tea shoots

**DOI:** 10.3389/fpls.2023.1224884

**Published:** 2023-07-18

**Authors:** Luyu Shuai, Ziao Chen, Zhiyong Li, Hongdan Li, Boda Zhang, Yuchao Wang, Jiong Mu

**Affiliations:** ^1^ College of Information Engineering, Sichuan Agricultural University, Ya’an, China; ^2^ Ya’an Digital Agricultural Engineering Technology Research Center, Sichuan Agricultural University, Ya’an, China; ^3^ College of Law, Sichuan Agricultural University, Ya’an, China; ^4^ College of Mechanical and Electrical Engineering, Sichuan Agricultural University, Ya’an, China

**Keywords:** dense small object detection, multimodal image fusion, RGB-D-IR, scale matching, frequency domain, attention mechanism, tea shoots

## Abstract

**Introduction:**

The difficulties in tea shoot recognition are that the recognition is affected by lighting conditions, it is challenging to segment images with similar backgrounds to the shoot color, and the occlusion and overlap between leaves.

**Methods:**

To solve the problem of low accuracy of dense small object detection of tea shoots, this paper proposes a real-time dense small object detection algorithm based on multimodal optimization. First, RGB, depth, and infrared images are collected form a multimodal image set, and a complete shoot object labeling is performed. Then, the YOLOv5 model is improved and applied to dense and tiny tea shoot detection. Secondly, based on the improved YOLOv5 model, this paper designs two data layer-based multimodal image fusion methods and a feature layerbased multimodal image fusion method; meanwhile, a cross-modal fusion module (FFA) based on frequency domain and attention mechanisms is designed for the feature layer fusion method to adaptively align and focus critical regions in intra- and inter-modal channel and frequency domain dimensions. Finally, an objective-based scale matching method is developed to further improve the detection performance of small dense objects in natural environments with the assistance of transfer learning techniques.

**Results and discussion:**

The experimental results indicate that the improved YOLOv5 model increases the mAP50 value by 1.7% compared to the benchmark model with fewer parameters and less computational effort. Compared with the single modality, the multimodal image fusion method increases the mAP50 value in all cases, with the method introducing the FFA module obtaining the highest mAP50 value of 0.827. After the pre-training strategy is used after scale matching, the mAP values can be improved by 1% and 1.4% on the two datasets. The research idea of multimodal optimization in this paper can provide a basis and technical support for dense small object detection.

## 1 Introduction

In recent years, the aging trend of agricultural labor has significantly intensified, and the difficulty in recruiting and expensive labor has limited the development of the tea industry (Han et al., 2014). The manual picking of premium tea accounts for about 60% of the labor used for managing the whole tea plantation, while excellent high-grade tea is picked with delicate leaf tips that grow in different positions, postures, and densities, making it difficult for machine picking especially in the unstructured environment with wind and light changes (Xu et al., 2022). Thus, it is essential to study intelligent tea-picking technology to promote the development of the tea industry. The key to realizing automated tea picking is the accurate identification of tea shoots. In recent years, with the development and application of computer technology, the accurate identification of tea shoots based on image processing has become a research hotspot (Lin et al., 2019).

Since there are obvious color differences between tea shoots and old leaves and tree trunks, color features can be used to extract shoot regions in the image, so the early research on tea shoot segmentation is mainly based on color features. The primary process of traditional image processing algorithms based on color space involves image pre-processing, color feature selection, segmentation, and other steps (Bojie et al., 2019). To further address the issue that tea leaf segmentation under natural conditions is easily affected by the external environment, such as old leaves, branches, and soil, and obscured and overlapping tea leave. Machine learning methods have been introduced for identification by extracting and synthesizing various feature sample data for training, and standard methods for tee shoot identification are developed based on features such as color, texture, and shape, combined with the use of K-mean clustering, support vector machine methods, Bayesian discriminant methods, and cascade classifiers. Recognition methods based on traditional machine vision rely on image pre-processing and data conversion, and unreasonable pre-processing will significantly affect the accuracy of the model (Karunasena and Priyankara, 2020) (Li et al., 2021).

The algorithm based on deep learning has high accuracy, providing a basis for studying intelligent tea shoot-picking equipment in complex backgrounds. To alleviate the influence of a complex environment on the performance of the detection model, (Xiaoxiao et al., 2019) employed a pre-segmentation method and then used the improved YOLO series of medium and large-scale network models to detect tea shoots with an average accuracy of 84.2%. To promote the deployment of models for detecting tea shoots to picking leaf tips, lightweight models have received much attention from researchers. (Xu et al., 2022) exploited the fast detection capability of YOLOv3 and the high-precision classification capability of DenseNet201 through a cascaded network to detect tea shoots accurately. Although the above methods have relatively high accuracy, robustness, and generalization performance, they are difficult to detect adequate tea shoots in complex environments on low arithmetic devices in farmland due to the high dependence of deep learning network models on arithmetic power. Thus, researchers have investigated the accuracy, speed, and lightness of model detection simultaneously (Cao et al., 2022). proposed a tea shoot detection algorithm that fuses GhostNet and YOLOv5; (Li Y. et al., 2022) designed a YOLOv3-SPP deep learning algorithm based on channel and layer pruning, which reduced the number of parameters, model size, and inference time while achieving efficient and accurate tea shoot detection. Note that few studies have focused on crop objects that are dense and minutely difficult. However, in the study of small target detection problems, remote sensing image target detection has achieved excellent results. (Wu et al., 2019) presented a detector called ORSIm, which effectively improves the accuracy of small target detection in optical remote sensing images by integrating different channel features, feature learning, and fast image pyramid matching and enhancement strategies. To reduce the difficulty in infrared small target detection, (Wu et al., 2023) proposed an interactive cross-notice nested U-Net network called UIU-Net. However, UIU-Net models infrared small target detection as a semantic segmentation problem, which increases the cost of labeling. Therefore, this study improves the detection performance of dense and tiny tea shoots by improving the target detection model and adopting migration learning techniques.

The above studies took only RGB images as the input to the network. Nevertheless, in an unstructured environment, a single sensor provides limited information to detect shoot targets under various difficulties, such as different lighting conditions, the similar color of tea shoots to the background, the small size of tea shoots, dense tea shoots, overlapping tea shoots, branch and leaf occlusion, as well as different poses. To overcome these difficulties, the approach of using multimodal data can be adopted since there is a certain complementarity and consistency between multimodal information. Although RGB images can reflect features such as color, brightness, and texture of objects, they can only provide two-dimensional (2D) details. With the further development of image acquisition devices, the availability of multimodal data for object detection in agricultural environments has increased greatly, such as depth images, infrared images, etc. (Sun et al., 2022). Depth images contain information about the distance from the object to the sensor, which can reflect the depth and three-dimensional (3D) morphology of the object. So, depth images have more unique edge features and shape features that can be exploited to better distinguish between foreground and background. Meanwhile, infrared images collect information about the heat distribution of the object, which can reflect the temperature and thermal radiation characteristics of the object. Most importantly, depth and infrared images are less affected by illumination and viewing angle, and they can be used to perform stable target detection in complex environments. Thus, in recent years, research work has been devoted to using multimodal information to improve the performance of crop detection. For instance, (Tao and Zhou, 2017) extracted improved 3D descriptors (Color-FPFH) that incorporate color features and 3D geometric features from pre-processed point clouds to obtain richer feature information to enhance the accuracy of detecting apples. (Gan et al., 2018) designed an algorithm for green citrus fruit detection by integrating image alignment, information fusion, fruit classification, and detection into a single step to realize real-time detection. Experimental results indicate that the fusion of color and thermal images can effectively improve the detection of unripe green citrus fruits. Additionally, some studies use depth information to exclude complex backgrounds in agricultural environments to enhance the detection performance of target objects in RGB images. For example, (Lin et al., 2019) presented a depth filter and Bayesian classifier-based image segmentation method based on red-green-blue-depth (RGB-D) images to remove complex backgrounds. This improves citrus detection and localization accuracy in a natural outdoor orchard environment. (Fu et al., 2020) developed a faster R-CNN-based apple detection method using RGB images and depth features in a dense leafy wall tree. The background was first eliminated using a depth threshold of 1.2 m to obtain the foreground RGB image. Then, the detection results of the original RGB image and the foreground RGB image were compared by using two different pre-trained network architectures (ZFNet and VGG16). The results demonstrated that removing the background tree using the depth filter can improve the fruit detection accuracy by 2.5%.

Methods for effective fusion methods of multimodal information have attracted much attention. In multimodal image target detection, the fusion methods for different information can be usually divided into three types: data layer fusion, feature layer fusion, and decision layer fusion. First, data layer fusion methods treat multimodal data as indistinguishable multichannel data and can exploit the inherent complementarity between different modalities to supplement the incomplete information in the input stage. For instance, (Gené-Mola et al., 2019) collected RGB images, depth images, and infrared images of apples simultaneously and performed range-correction on the signal intensity to solve the signal attenuation problem. The detection of apples was achieved by applying the Faster R-CNN model to five channels of input images (color (RGB), depth (D), and distance-corrected intensity signal (S)). The results indicate that the F1-score improves by 4.46% when depth and range-corrected intensity channels are added, and an F1-score of 0.898 and an AP of 94.8% are obtained when all channels are used. (Liu et al., 2019) proposed a method to fuse aligned RGB images, NIR images, and deep convolutional neural networks for kiwifruit detection. In their study, two different fusion methods were investigated: image fusion (fusing RGB and infrared images on the input layer) and feature fusion (combining the feature maps of two VGG16 networks with separate input RGB and NIR images). The results showed that the highest AP value of 90.7% was achieved by using the image fusion method. (Rong et al., 2023) applied a multimodal (RGB images and depth images) data fusion approach to optimize the input of YOLOv5 to reduce the effect of background on false tomato recognition and improved the recall of unripe tomatoes with a detection accuracy of 97.9% by the improved YOLOv5-4D. However, the crude data layer fusion method may result in information redundancy and noise propagation with limited enhancement effect, affecting the quality and accuracy of the fused data. The second type of fusion method, i.e., the feature layer fusion method, inputs multimodal images into parallel branches, extracts independent features at different scales in different modes, and then fuses the features. For instance, (Wu et al., 2021) developed a new multimodal remote sensing image classification network called CCR-Net. CCR-Net uses features from different modalities obtained by a CNN extractor and fuses them more compactly, allowing better processing and analysis of multimodal remote sensing data. (Hong et al., 2021) designed a new supervised algorithm for GCNs, called miniGCNs. miniGCNs jointly uses CNNs and GCNs to extract more diverse and differentiated feature representations for hyperspectral image classification tasks. However, both are based on image classification tasks. (Sun et al., 2022) proposed a noise-tolerant RGB-D feature fusion network for outdoor fruit detection to integrate RGB feature information, depth feature information, and an attention-based fusion module to adaptively fuse multimodal features to remove the adverse effects of depth noise and focus perception on the essential parts of the features. The proposed NT-FFN achieves an AP50 value of 95.4%. However, the inappropriate feature fusion approach in the feature layer fusion method may increase the difficulty of model learning and aggravate the imbalance of the network learning modality. The third type of feature fusion method, i.e., the decision layer fusion method, fuses the detection results of the last stage. For example, (Tu et al., 2018) adopted a faster region-based convolutional neural network (Faster R-CNN) to detect passion fruit for color images and depth images, respectively, and the two detection results based on RGB images and depth images were combined to improve the detection performance. (Lin et al., 2022) developed a regression network with multi-branch architecture to extract and fuse RGB, depth, and geometric features easily. The proposed post-fusion architecture significantly improved the fresh weight detection accuracy of lettuce shoots at different growth periods. However, the decision-level fusion method may consume a lot of computational resources due to the repeated computation of other multimodal branches, and the process learns the features of individual modalities independently without considering the correlation between different modal information. Therefore, to realize efficient real-time detection of tea shoots in an agricultural intelligent picking environment, this study investigates two data layer-based multimodal information fusion methods and a feature layer-based multimodal information fusion method, respectively. Meanwhile, a lightweight frequency domain attention mechanism module is designed for the feature layer fusion method to effectively fuse feature information across modalities.

To efficiently detect small targets of dense tea shoots in complex environments, this study improves the architecture of the YOLOv5 target detection model. Additionally, to make up for the deficiency of RGB image-based tea shoot detection, this study designs two data layer-based multimodal fusion methods and a feature layer-based multimodal fusion method based on the YOLOv5 model and designs a cross-modal fusion module based on frequency domain and attention mechanism. The main contributions of this study are summarized below:

1. A tea image dataset of the natural environment is constructed. It contains aligned RGB images, depth images, and infrared images; the RGB images are annotated with tea shoot objects.
2. The architecture of the YOLOv5 model is modified and adjusted to improve the detection performance of the model for dense and tiny tea shoots.
3. The scale matching method is optimized based on the object scale. The generalization and robustness of the tea shoot detection model are improved by applying transfer learning techniques.
4. Two multimodal fusion methods based on the data layer and one multimodal fusion method based on the feature layer are investigated. Meanwhile, a cross-modal fusion module based on frequency domain and attention mechanism is designed to learn complementary information by adaptively focusing key regions in intra- and inter-modal frequency domain dimension and channel dimension to improve the performance of the tea shoot detector.

## 2 Materials and methods

### 2.1 Data

#### 2.1.1 Data acquisition

The dataset used in this study was obtained at the National Tea Tree Breeding Farm, Mengdingshan Tea Modern Agricultural Park, Ya’an City, Sichuan Province, China. The images were taken on the evening of 09/03/2023 and 19/03/2023, the prime time for famous tea harvesting. This study took Microsoft Kinectv2 as the image acquisition device, which integrates an RGB camera and a depth sensor that works following the TOF principle. The sensor provides three types of data: a color image, a depth image that can generate a 3D point cloud of the scene, and a received infrared backscattered intensity image.

In the data acquisition process, the Microsoft Kinect v2 depth camera was fixed on a triangular stand, with one end of the camera being connected to 220V outdoor mobile power and the other end being connected to a laptop *via* USB 3.0. The depth image, infrared image, color, and depth information aligned low-resolution image were captured simultaneously on the computer by calling the API of PyKinectV2 (Kinect/PyKinect2). First, a depth image, an infrared image, and an aligned image (RGB) with both color and depth information were captured simultaneously; then, they were resized to 512×424 pixels; finally, the images were mirrored and inverted separately and saved. The RGB image was stored in 24 bits, the infrared image in 16, and the depth image in 8. The depth camera was placed vertically from 0.5-1.0 m away from the top of the tea. To reduce the effect of bright light on sensor performance under outdoor conditions, all data were captured from 5:00 to 7:00 PM on an overcast day. **Table 1** presents the parameters and specifications of the equipment used in the data acquisition process.

**Table 1:** Acquisition equipment specifications.

Device	Specifications	Parameter
RGB-D Sensor	Manufacturer and model	Microsoft Kinectv2
	RGB channel resolution (pixels)	1920 × 1080
	RGB channel field-of-view (FOV)	84.1° × 53.8°
	IR and Depth channel resolution (pixels)	512 × 424
	IR and Depth channel FOV	70° × 60°
	Working range (m)	0.5–8
Notebook Computer	Manufacturer and model	ASUS
	Processor	AMD Ryzen 7 6800H with Radeon Graphics 3.20 GHz
	RAM	16.0 GB
Outdoor mobile power	Manufacturer and model	St. Xinlong
	Size	255×165×145mm
	Power capacity	90000mAh
	Output voltage	220V

#### 2.1.2 Data preparation

**Figure 1:**
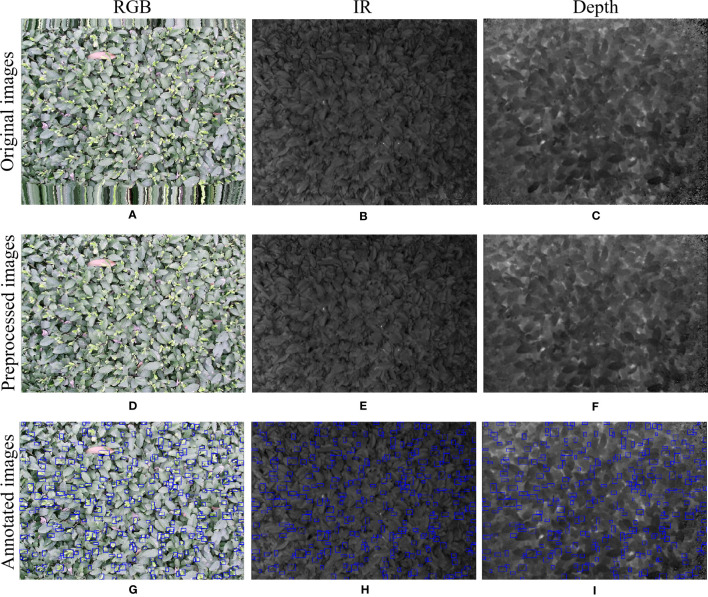
RGB images, IR images, and Depth images are represented from left to right. **(A-C)** captured original image; **(D-F)** cropped image; **(G-I)** annotated image. .

A multimodal image dataset consisting of RGB, infrared, and depth images was obtained after data acquisition, each with a resolution of 512×424 pixels. The original image schematic is shown in the first row of **Figure 1**. Since the depth sensor has a larger vertical field of view than the color camera, the RGB, infrared, and depth images were cropped by removing the bottom and top images that do not provide RGB information, and the image resolution became 521×360 pixels, as shown in the second row of **Figure 1**.

In the data annotation process, tea shoots were manually annotated using the COCO Annotator (Stefanics et al., 2022) online annotation software for RGB images only. To simulate the complexity of tea shoot growth in a natural environment and reflect the effectiveness of the detector, tea shoots with less than 75% occlusion and tiny tea shoots were annotated with absolute pixels larger than 2×2 pixels. Each image annotation process took 0.5-0.6 hours, and each image contains 200-400 tea shoot targets with an absolute scale of about 30×30 pixels. To achieve a low manual annotation cost and investigate the effect of multimodal images on the performance of tea shoot detection, RGB, infrared, and depth images shared a common set of labels: the annotation result on RGB images. An example of the image after mapping the labeling results to infrared and depth images is shown in the third row in **Figure 1**.

This study collected 100 sets of multimodal image data on 09/03/2023 and 19/03/2023, respectively, 200 sets in total. Each dataset contains one RGB, infrared, and depth image, as well as the corresponding labels. **Table 2** shows the distribution of the datasets and example images. Dataset1 and Dataset2 represent the datasets collected on 09/03/2023 and 09/03/2023, respectively. Dataset 3 represents the set of Dataset 1 and Dataset 2 datasets.

**Table 2:** Distribution of data sets and image examples.

Datasets		Collection time	Number	RGB	IR	Depth	Label
Dataset3	Dataset1	2023.03.09	100	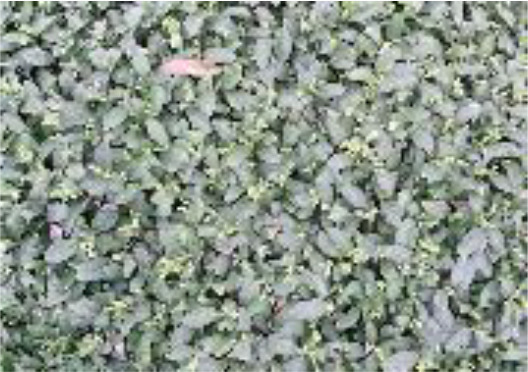	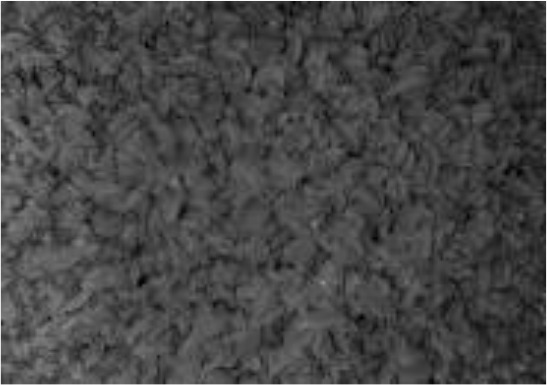	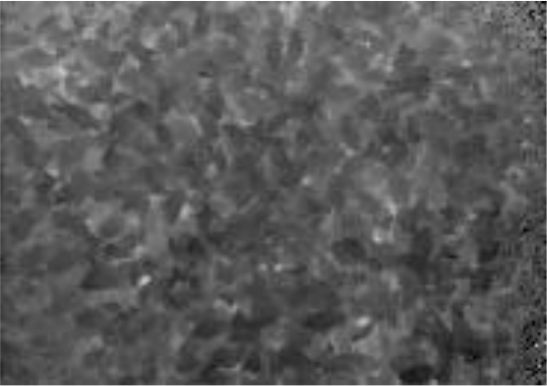	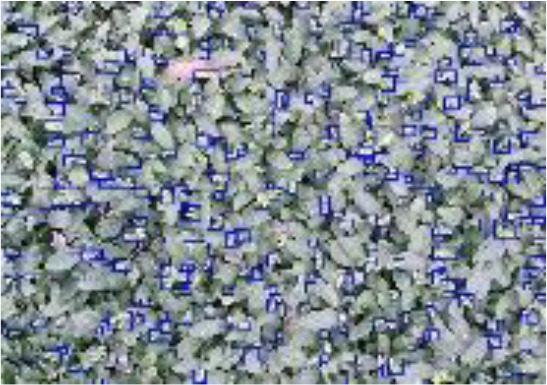
	Dataset2	2023.03.19	100	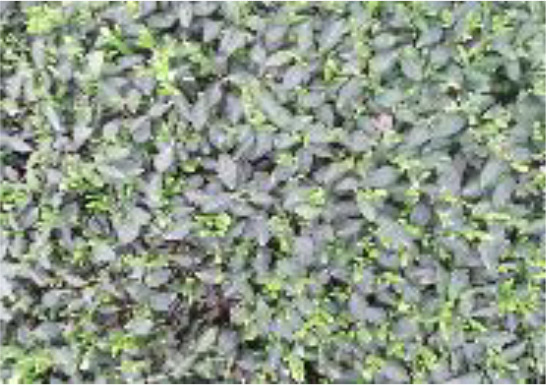	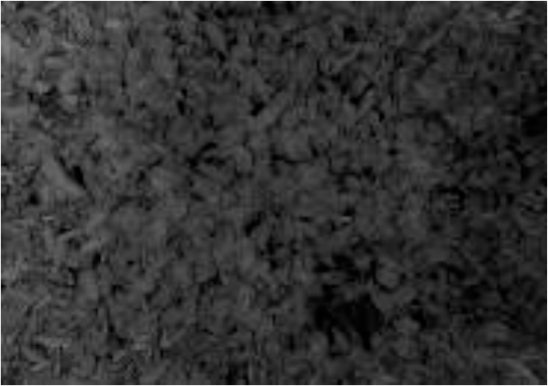	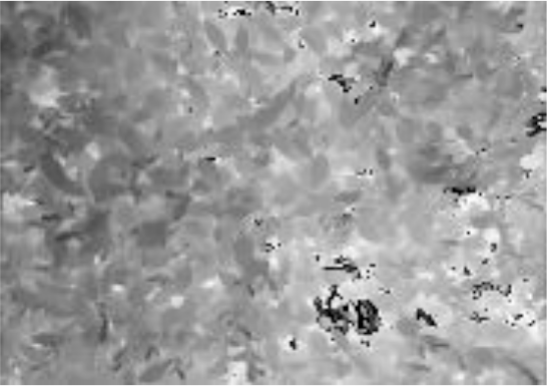	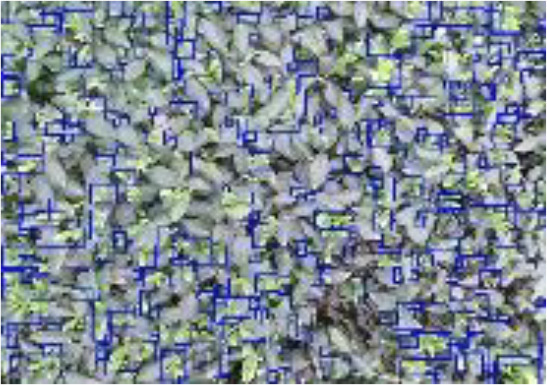

Since the color camera has a more significant horizontal field of view than the depth sensor, the original high-resolution color image (1920×1080 pixels) and the RGB image (521×360 pixels) used in this study were unaligned, and this study aimed to investigate the detection method and model for dense small targets in low-resolution images. Therefore, this study only used the low-resolution RGB images and the aligned infrared and depth images as experimental data. In future work, we will explore the problem of image alignment and super-resolution-assisted small target detection based on high-resolution and low-resolution images, and the original high-resolution color images will be used.

### 2.2 Methods

#### 2.2.1 YOLOv5s baseline and improvement architecture

**Figure 2:**
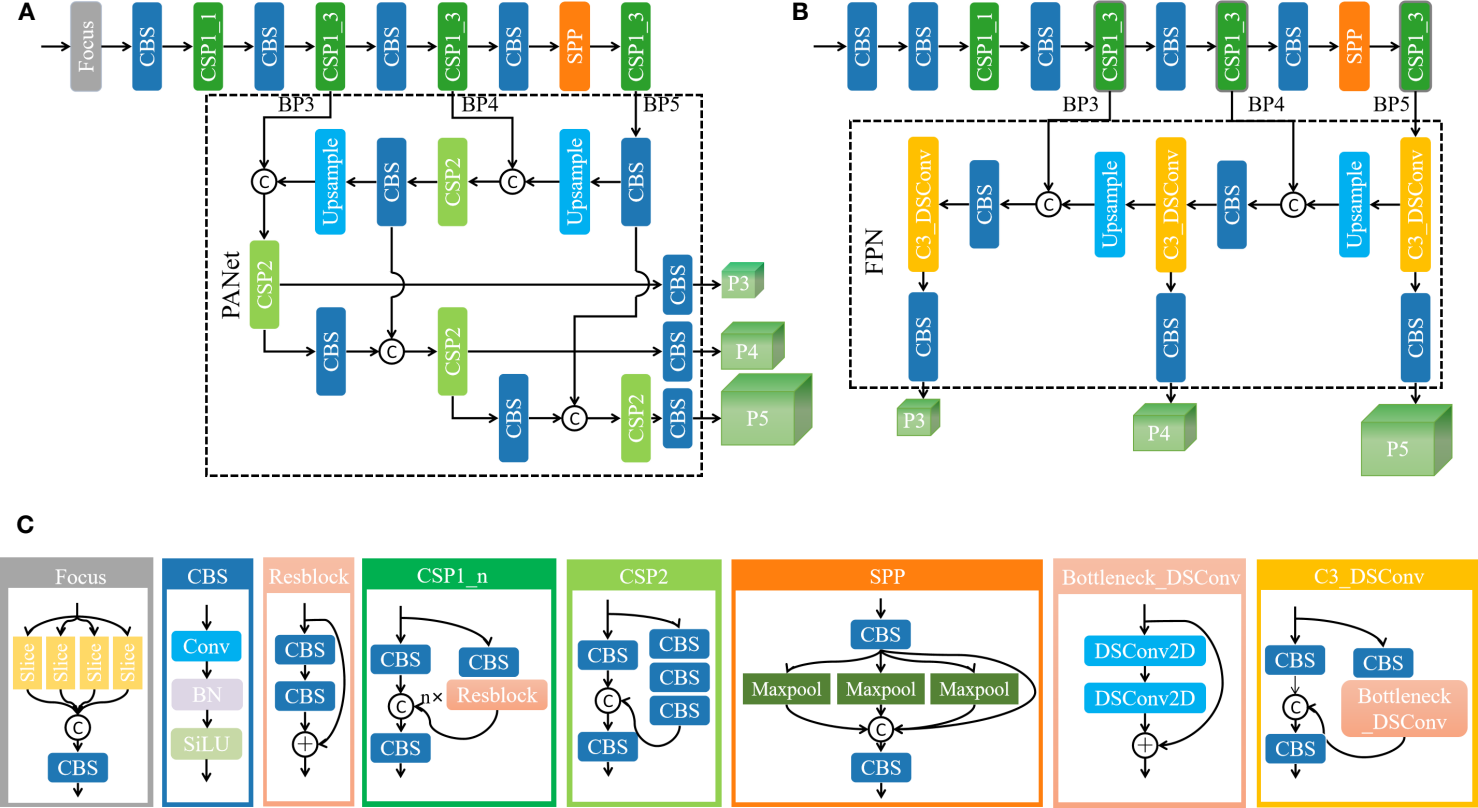
Model architecture diagram and detailed module construction diagram. **(A)** YOLOv5s model architecture diagram; **(B)** YOLOv5s_improve model architecture diagram; **(C)** detailed construction of the modules that may be included in the model.

YOLO (You Only Look Once) (Redmon et al., 2016) is a classic single-stage target detection network. The YOLOv5 (Jocher et al., 2022) model is widely used in various target detection tasks because of its flexibility and versatility. It uses CSPNet (Cross Stage Partial Network) (Wang et al., 2020) as the backbone to extract feature information and SPP (Spatial Pyramid Pooling) (He et al., 2015) to extract multi-scale depth features and then fuse the features at different scales through a feature pyramid constructed by PANet (Path Aggregation Network) (Liu et al., 2018), and the final results are output through three detection heads P3, P4, and P5. The depth and width of the YOLOv5 model depend on the bottleneck layer and several convolutional kernels, whereas the YOLOv5s model has a small size and fast inference speed, which is beneficial for real-time target detection in realistic scenarios. This is the reason why this study chooses YOLOv5s as the baseline. However, since the baseline model is usually designed for detecting medium and large targets, there are some limitations in the detection of small objects. YOLOv5s mainly includes the Focus layer, the design of the CSP1_n module, the number of stacks, and the PANet architecture. This study will elaborate on their limitations and the corresponding improvement measures for dense and tiny tea shoot detection. **Figures 2A, B** show the architectures of the YOLOv5 model and our improved YOLOv5s_improve model, respectively, and **Figure 2C** shows the detailed construction of the modules that may be included in these two models.

The limitations and improvements are analyzed as follows:

1. From Focus to Conv: Focus is a lightweight convolutional layer. To reduce computational cost and speed up network training and inference, the Focus layer divides the input into four parts; convolutional operations are performed on each part separately, and the results are stacked finally to form the output feature map. However, this approach may sacrifice the accuracy of small target detection. Therefore, to better capture the feature information of small targets, this study uses replaces the Focus layer with a superficial Conv layer to increase the perceptual field of the model and the feature representation.
2. From “3693” to “8833”: The backbone of YOLOv5 used convolution with a step size of 2 in the early stage to halve the feature size. As the network deepens, the feature size retained for multi-scale target detection is much smaller than the size of the original input image. This low-resolution feature map does not contain information that can be used to reliably distinguish tiny objects. (Ning et al., 2023) effectively improved the performance of small object detection by increasing the shallow layers (the convolutional layers in the high-resolution stage) in the ResNet (He et al., 2016) and HRNet (Sun et al., 2019), thereby using fewer convolutional layers in the later stages of the network. The experimental results indicated that the early downsampling leads to information loss and difficulty in representing the features of small targets. Similarly, the number of CSP1_n modules in each phase of the YOLOv5 backbone network is modified to allocate more resources to handle higher-resolution features, and the number of CSP1_n modules in the post-backbone stage of the network is reduced to not introduce additional computational burden. The original YOLOv5 backbone contains four CSP1_n modules, and the number of modules is 3, 6, 9, and 3 in order. Through several experimental adjustments, this study finds that the optimal number of CSP1_n modules is 8, 8, 3, and 3 in order.
3. From CSP2 to C3_DSConv: In the CSP2 module of the neck, the standard convolution operation may cause the small object model of tea shoots to overfit and introduce an enormous computational burden. (Nascimento et al., 2019) proposed a flexible quantized convolution operator DSConv that uses inexpensive integer operations instead of single-precision operations while maintaining the kernel weights and output on the probability distribution. This study replaces the standard convolution in the neck CSP2 module with DSConv to ensure the lightweight and real-time characteristics of the tea shoot detection model.
4. From PANet to FPN: The main idea of PANet is to obtain higher-level semantic information through aggregation and transfer, but it requires a lot of computational resources and time and may lead to information loss and model overfitting, and PANet focuses on the improvement of detection accuracy of medium and large targets. FPN (Feature Pyramid Network) (Lin et al., 2016) obtains better scale adaptation and semantic information through feature transfer and fusion, which helps to preserve the delicate features and information required for small object detection and effectively reduces the complexity of the model. Thus, this study replaces the PANet structure with FPN.

#### 2.2.2 Multimodal object detection architecture

##### 2.2.2.1 Multimodal image object detection

**Figure 3:**
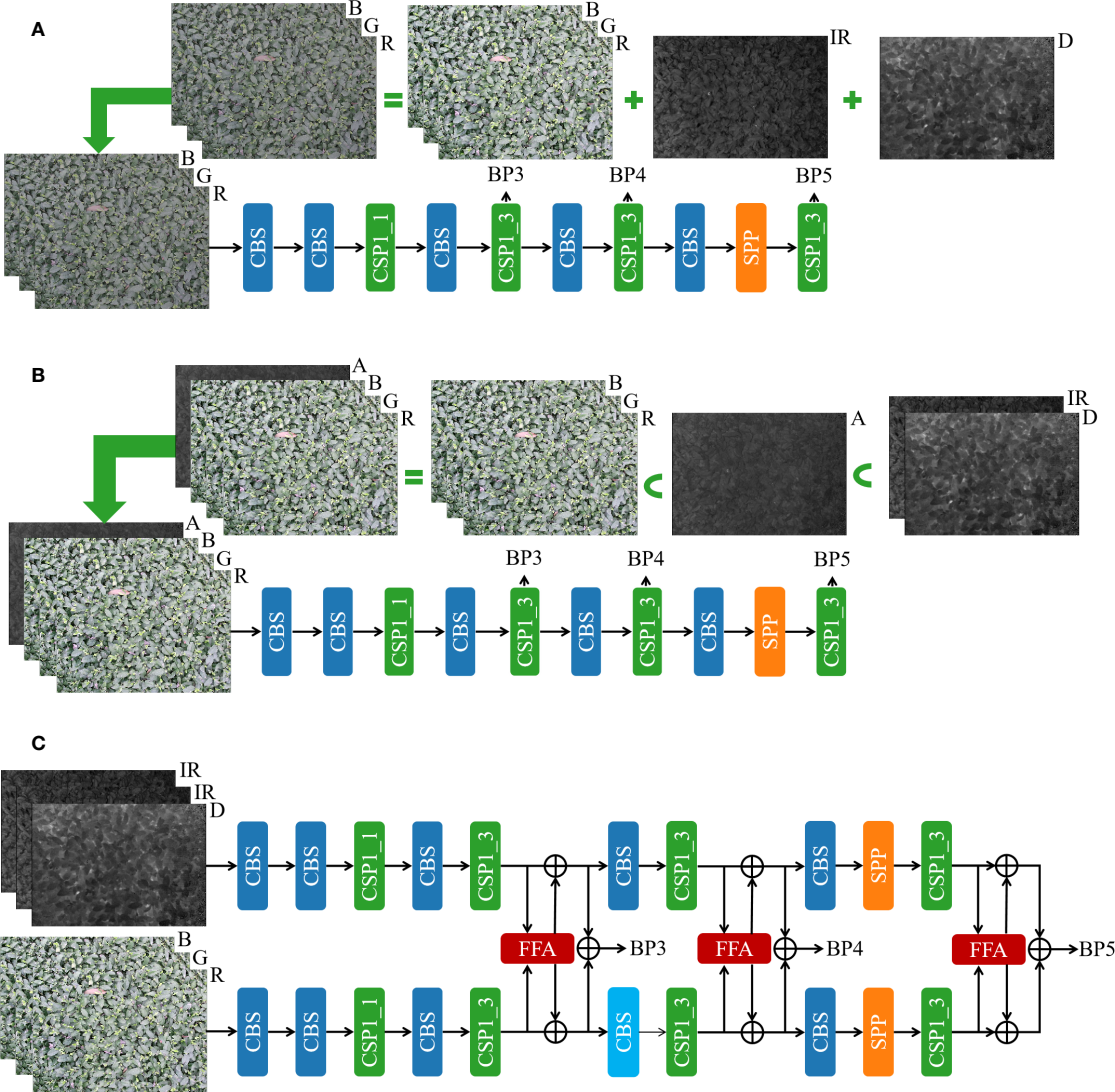
Three fusion methods for multimodal images. **(A)** Data layer-based fusion method 1; **(B)** Data layer-based fusion method 2; **(C)** Feature layer-based fusion method.

To fully utilize the complementary information between RGB, infrared, and depth images of tea shoots to enhance the ability of the model to detect and localize tea shoots, two data layer-based fusion methods and a feature layer-based fusion method is established in this study. Besides improving the quality of intra-modal and inter-modal information fusion, a simple and effective FFA module is designed in this study by using the feature layer-based data fusion method. The input and the backbone of the models of the three fusion methods in this study are illustrated in **Figure 3**.

Method 1 uses a simple data layer fusion approach. As shown in method (A) in **Figure 3**, through several repetitive comparative experiments, the best weighting coefficients are first derived for RGB, infrared, and depth images, and they are 0.6, 0.2, and 0.2, respectively. Secondly, the RGB, infrared, and depth images are fused by simple pixel-level summation with the best weighting coefficients, respectively. Then, the synthesized images are fed into the single-stream object detection backbone for feature extraction. Finally, BP3, BP4, and BP5 features are provided to the model head for detection.

Method 2 uses data layer fusion based on channel mapping. Again, the best weighting coefficients are derived for infrared and depth images by repeated experiments with multiple comparisons of 0.5 and 0.5, respectively. Then, the infrared and depth images are fused by simple pixel-level summation with the best weighting coefficients. The obtained image A is taken as the fourth channel of the image to obtain a four-channel RGBA image by stitching it with the color RGB image. Next, the RGBA image is fed into the designed 4-channel single-stream object detection backbone for feature extraction, and finally, BP3, BP4, and BP5 features are provided to the model head for detection. The details are shown in method (B) in **Figure 3**.

Method 3 uses feature layer fusion. The infrared and depth images are first stitched into a single three-channel image (D_IR_IR) to preserve as much information as possible under each modality; then, the stitched and colored RGB images are fed into the designed dual-stream object detection backbone to extract features, and finally, BP3, BP4, and BP5 features are provided to the model head for detection. The detailed design of YOLOv5s-Multimodal, a multimodal image fusion architecture based on feature layers, is presented in **Figure 3C**. In the YOLOv5s_Multimodal model, this study uses YOLOv5s_improve as the backbone of two branches, but the parameters in the two backbones are not shared. The same backbone structure is used to extract features from D_IR_IR and RGB images under each modality. In the intermediate stage of the backbone, the features are fused by the frequency domain-based cross-modal fusion attention module (FFA) to facilitate the interaction and fusion of modalities, and the fused features are fed to the RGB stream and the D_IR_IR stream respectively for feature extraction in depth.

##### 2.2.2.2 Cross-modal fusion attention module based on frequency domain

**Figure 4:**
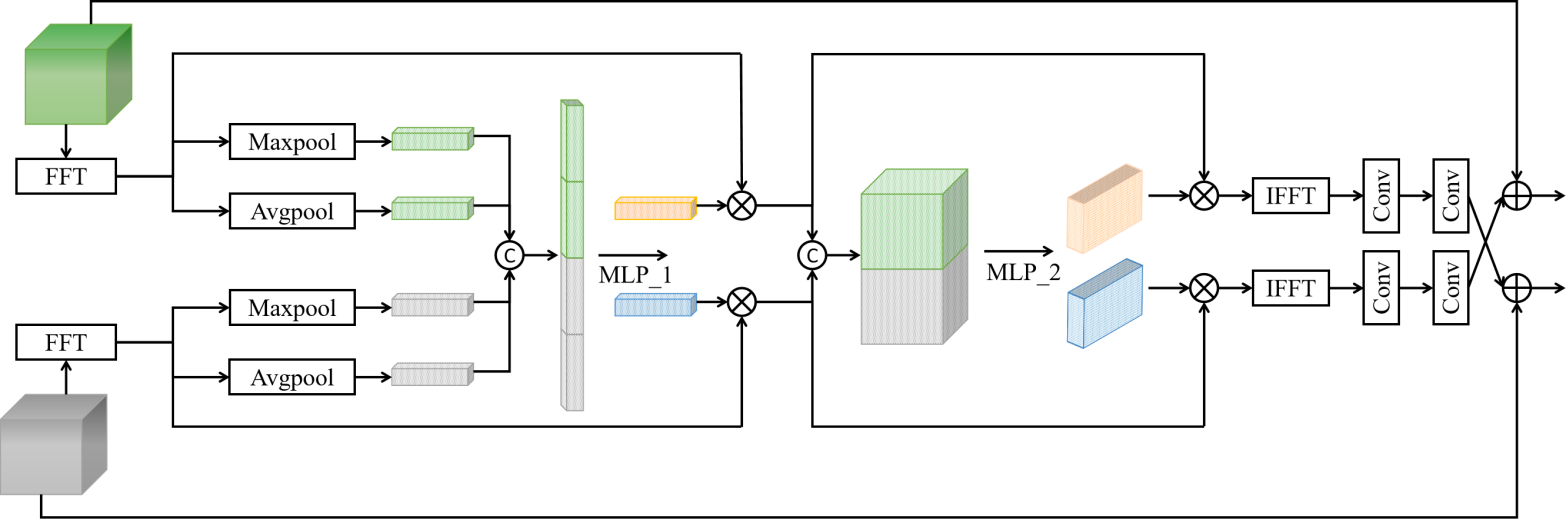
Structure of the FFA module.

RGB, infrared, and depth images have their strengths and weaknesses, and their information is usually complementary but contains noise. There are better solutions than simply fusing or processing RGB, infrared, and depth images. However, noisy information can be filtered and calibrated using features from another modality, so this study proposes FFA, and its structure is shown in **Figure 4**.

To reduce expensive computations, improve the inference speed of the model and better preserve the spatial and semantic information of the images, this study chooses to filter, enhance, and fuse the information of different modalities in the frequency domain. To resolve the noise and uncertainty in other modalities and to calibrate and extract the frequency feature information in various modalities, this study infers the attention map along the channel dimension and frequency dimension in turn and then multiplies the attention map with the feature map in the frequency domain to perform adaptive frequency domain feature fusion optimization. To facilitate feature extraction and interaction between modes, this study enhances information interaction between other methods by simple convolution and cross-fusion.

Spatial domain to frequency domain: feature maps $F_{rgb}$ and $F_{depth\_ir}$ are respectively converted to $F_{f\_rgb}$ and $F_{f\_depth\_ir}$ in the frequency domain using FFT. Equations (1-2) show the corresponding 2D FFT.


(1) $$F_{f\_rgb}\left(u,v\right)=\sum_{x=0}^{M-1}\sum_{y=0}^{N-1}F_{rgb}\left(x,y\right)e^{-j2\pi \left(\frac{ux}{M}+\frac{vy}{N}\right)}$$

(2) $$F_{f\_depth\_ir}\left(u,v\right)=\sum_{x=0}^{M-1}\sum_{y=0}^{N-1}F_{depth\_ir}\left(x,y\right)e^{-j2\pi \left(\frac{ux}{M}+\frac{vy}{N}\right)}$$

where $F\left(x,y\right)$ is a feature map of size M×N , and equations (1) and (2) are evaluated for the discrete variables *u* and *v* with u=0,1,2,…,M−1 and v=0,1,2,…,N−1 .

Information fusion and enhancement of channel dimensions: First, global pooling operations are performed on the frequency -domain feature maps $F_{f\_rgb}$ and $F_{f\_depth\_ir}$ respectively to obtain global frequency -domain feature information, and both global average pooling and global maximum pooling are used to retain as much information as possible. Then, four resultant vectors are generated and stitched to form a richer frequency -domain feature representation. Next, the frequency -domain feature information is further extracted and fused by the MLP_1 layer. Subsequently, the sigmoid operation is performed to obtain the weights, and the weights are divided into $W_{f\_rgb}^{C}$ and $W_{f\_depth\_ir}^{C}$ by the split operation. Finally, the weights are multiplied with the input frequency-domain feature maps $F_{f\_rgb}$ and $F_{f\_depth\_ir}$ to obtain the frequency-domain feature maps $F_{f\_rgb}^{C}$ and $F_{f\_depth\_ir}^{C}$ , respectively. In this way, the information enhancement and complementation of the channel dimension of RGB and Depth_IR features are realized. The whole process is shown in Equations (3-7).


(3) $$F_{f\_rgb\_c}=Concat((MaxPool(F_{f\_rgb})),(AvgPool(F_{f\_rgb})))$$

(4) $$F_{f\_depth\_ir\_c}=Concat((MaxPool(F_{f\_depth\_ir})),(AvgPool(F_{f\_depth\_ir})))$$

(5) $$W_{f\_rgb}^{C}\;,W_{f\_depth\_ir}^{C}=F_{split}(\delta (F_{MLP\_1}(Concat(F_{f\_rgb\_c}\;,F_{f\_depth\_ir\_c}))))$$

(6) $$F_{f\_rgb}^{C}=W_{f\_rgb}^{C}⊛F_{f\_rgb}$$

(7) $$F_{f\_depth\_ir}^{C}=W_{f\_depth\_ir}^{C}⊛F_{f\_depth\_ir}$$

where δ represents the Sigmoid operation.

Information fusion and enhancement in the frequency domain: first, the Concat operation is performed on frequency -domain feature maps $F_{f\_rgb}^{C}$ and $F_{f\_depth\_ir}^{C}$ to obtain a richer frequency -domain feature representation. Then, after MLP_2 layers, which are two 1×1 convolution and nonlinear transform RELU operations, more features are extracted to obtain a complex frequency -domain feature representation. Next, the sigmoid operation is performed to obtain the weights, and the weights are divided into $W_{f\_rgb}^{F}$ and $W_{f\_depth\_ir}^{F}$ by the split operation. Finally, the weights are multiplied with the input frequency-domain feature maps $F_{f\_rgb}^{C}$ and $F_{f\_depth\_ir}^{C}$ to obtain the frequency-domain feature maps $F_{f\_rgb}^{F}$ and $F_{f\_depth\_ir}^{F}$ , respectively. In this way, the information enhancement and complementarity of the frequency dimension of RGB and Depth_IR features are realized. The whole process is shown in Equations (8-11).


(8) $$F_{f\_rgb\_depth\_ir}^{C}=Concat\left(F_{f\_rgb}^{C}\;,F_{f\_depth\_ir}^{C}\right)$$

(9) $$W_{f\_rgb}^{F}\;,W_{f\_depth\_ir}^{F}=F_{split}(\delta (Conv_{1\times 1}(RELU(Conv_{1\times 1}(F_{f\_rgb\_depth\_ir}^{C})))))$$

(10) $$F_{f\_rgb}^{F}=W_{f\_rgb}^{F}⊛F_{f\_rgb}^{C}$$

(11) $$F_{f\_depth\_ir}^{F}=W_{f\_depth\_ir}^{F}⊛F_{f\_depth\_ir}^{C}$$

where δ represents the Sigmoid operation.

Frequency domain to spatial domain: IFFT is performed on feature maps $F_{f\_rgb}^{F}$ and $F_{f\_depth\_ir}^{F}$ to convert them back to feature maps $F_{rgb}^{CF}$ and $F_{depth\_ir}^{CF}$ in the spatial domain, respectively. The corresponding 2D IFFT is shown in Equations (12-13).


(12) $$F_{rgb}^{CF}\left(x,y\right)=\frac{1}{MN}\sum_{u=0}^{M-1}\sum_{v=0}^{N-1}F_{f\_rgb}^{F}\left(u,v\right)e^{j2\pi \left(\frac{ux}{M}+\frac{vy}{N}\right)}$$

(13) $$F_{depth\_ir}^{CF}\left(x,y\right)=\frac{1}{MN}\sum_{u=0}^{M-1}\sum_{v=0}^{N-1}F_{f\_depth\_ir}^{F}\left(u,v\right)e^{j2\pi \left(\frac{ux}{M}+\frac{vy}{N}\right)}$$

where x=0,1,2,…,M−1 , y=0,1,2,…,N−1 .

Re-enhancement of purified information: To obtain a better feature representation, two convolution operations are used to enhance the feature information extracted in the above process, and the information is fed to the RGB stream and Depth_IR stream respectively for the next stage of feature extraction and fusion by cross-fusion. Equation (14-15) shows the purified information re-enhancement operation.


(14) $$F_{rgb}=Conv_{1\times 1}(Conv_{1\times 1}(F_{depth\_ir}^{CF}))⊕F_{rgb}$$

(15) $$F_{depth\_ir}=Conv_{1\times 1}(Conv_{1\times 1}(F_{rgb}^{CF}))⊕F_{depth\_ir}$$

#### 2.2.3 Objective-based scale matching

**Figure 5:**
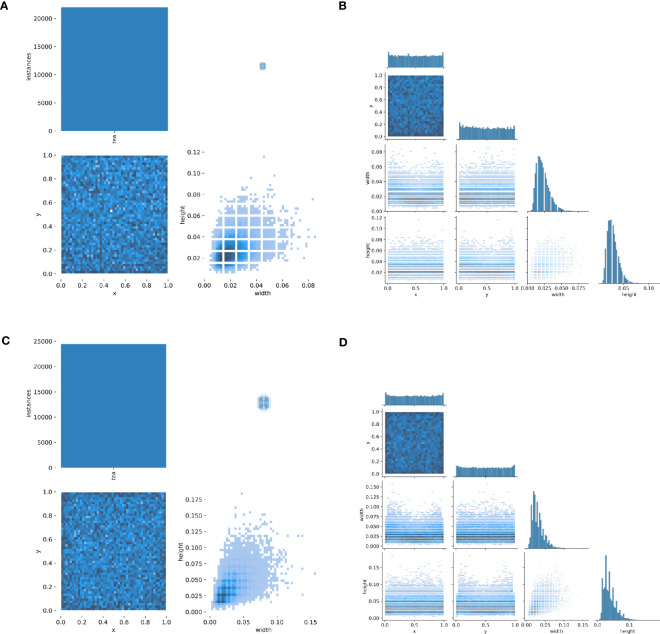
Distribution of tea shoot objects in Dataset1 and Dataset2 datasets. **(A)** the total number of targets and the relative width and height scales of target boxes in Dataset1; **(B)** the total number of objects and the relative width and height scales of object boxes in Dataset2; **(C)** the relative width and height scales and distribution of objects in Dataset1; **(D)** the relative width and height scales and distribution of objects in Dataset2.

The influence of uncontrollable factors in the natural environment, such as light, temperature, and humidity, leads to different growth states of tea shoots. Particularly, tea shoots proliferate from early March to early April, as shown in Dataset1 and Dataset2, which exhibit large differences in length, volume, posture, and color, although only ten days. This poses a challenge to the generalizability and robustness of the detection model. **Figure 5** shows the number and relative scale distribution of tea shoot objects in the two datasets.

From **Figure 5**, it can be observed that: In Dataset1, the total number of tea shoots exceeds 20,000, the distribution of tea shoots is relatively dense, the width and height of tea shoots are similar in attitude, and the relative scale of over 90% of the tea shoots is less than 5%. In Dataset2, the total number of tea shoots is close to 25,000, the distribution of tea shoots is very dense, the width, height, and posture of tea shoots are different, and the relative scale of over 90% of tea shoots is less than 10%. Overall, both datasets are dense, making it challenging to find targets. The difference between them is that Dataset1 has fewer samples and more minor relative scale differences, while Dataset2 has more samples and larger relative scale differences.

(Yu et al., 2020) found that the problem of scale mismatch reduces the accuracy of feature representation and detection models, and a smaller dataset may lead to model overfitting. To improve the generalization and robustness of the detector for detecting tea shoots of different periods under the condition of small samples, this study uses a simple scale-matching method combined with migration learning techniques to improve the detection performance of the model. The targets in Dataset2 are scaled to align with the relative scales of the targets in Dataset1. Then, the best weights obtained from training using the aligned dataset are used as pre-training weights to guide the detection model to fine-tune the parameters on Dataset1 to improve the detection capability of the detector for Dataset1. This facilitates the distribution of features between the pre-trained dataset of the aligned network and the dataset learned by the detector, enabling the model to better utilize the information at small scales.

**Figure 6:**
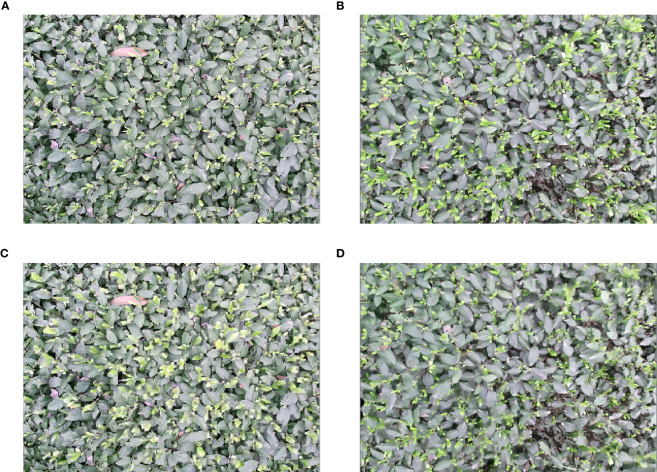
Objective-based scale matching method. **(A)** Example image in Dataset1; **(B)** Example image in Dataset2; **(C)** Example image after Dataset1 is aligned to Dataset2 scale; **(D)** Example image after Dataset2 is aligned to Dataset1 scale.

The specific procedure is as follows: first, the average scale (s_1_, s_2_) of the two datasets Dataset1 and Dataset2, and their distributions are calculated by statistical data methods, and the scale scaling factors (a_12_, a_21_) between the two datasets are obtained. Then, search, judgment, and scaling operations are performed for all targets in the images. For instance, for Dataset2, if the relative scale of an object is larger than the average scale s1, the target object is keyed out according to the label box, followed by scaling the object according to the scale scaling factor a_21_, and then the object is put back to the original position to keep the center position unchanged. Additionally, to not damage the contextual structure information of the target object, this study uses the adjacent pixel-based image interpolation method to recover the empty part caused by scaling the target object, and the same processing is conducted for Dataset1. **Figure 6** shows the image comparison effect of the objective-based scale matching method.

#### 2.2.4 Loss function

The loss function used to detect tea shoots in this paper consists of three components: confidence loss function, classification loss function, and boundary regression prediction loss function, as shown in Equation (16).


(16) $$L_{O}=L_{Confidence}+L_{Classification}+L_{Box}$$

## 3 Results and discussion

### 3.1 Experimental details

The experiment was conducted on a computer running Windows 10 operating system, and the hardware and software parameters are listed in **Table 3**. The official YOLOv5 version 6.1 (Jocher et al., 2022) codebase was taken, and the modifications described in sections 2.2.1 and 2.2.2 were implemented on top of it. The training was performed using the SGD optimizer. The initial learning rate was 1E-2, the final learning rate was 1E-5, and the weights decayed to 5E-3. After a momentum of 0.8 was used in the first three warm-up phases, it became 0.937. The training process was run for 300 epochs with a batch size of 4. Online data enhancement methods such as horizontal flip, random rotation, color change, and mosaic, were used during the training to enhance the sample diversity.

**Table 3:** Software and hardware parameters.

Accessories	Model
Operating system	Windows 10
CPU	Intel(R) Xeon(R) Gold 5218 CPU @ 2.30GHz
RAM	128 GB
GPU	NVIDIA Quadro RTX 5000
Development environments	Python3.8, Pytorch1.10.1, CUDA10.2

### 3.2 Evaluation metrics

In this study, floating point operations per second (GFLOPs), precision (Precision), recall (Recall), and average precision (mAP) were taken as evaluation metrics for measuring model complexity and performance. The calculation formulas of these metrics are shown in Equations (17-21).


(17) $$Precision=\frac{TP}{TP+FP}$$

(18) $$Recall=\frac{TP}{TP+FN}$$

(19) $$mAP=\frac{\sum_{1}^{N}AP}{N}=\frac{\sum_{1}^{N}\int_{0}^{1}P\left(R\right)dR}{N}$$

(20) $$Parameter=C_{in}\times C_{out}\times K\times K$$

(21) $$GFLOPs=(2C_{in}K^{2}-1)\times H_{out}W_{out}C_{out}$$

The parameter denotes the number of parameters of the model. GFLOPs is a metric of the computational power of the model, and a smaller GFLOPs value indicates that the model has less computational burden and can respond to requests faster. The two metrics visually represent the complexity of the model. TP, FP, and FN denote the number of correctly detected objects, incorrectly detected objects, and undetected tea shoot objects, respectively. Precision is the probability that a tea shoot is predicted to be a positive sample among the actual positive samples. The recall is the probability of tea shoots being predicted as positive among the actual positive samples. AP represents the average precision, a combination of precision and recall. The mAP is the average of AP of different categories, where N is the number of types; in this experiment, there is only one category of tea shoots, so N is 1. In this study, mAP50 and mAP95 refer to the mAP values when the value of IOU is taken at 50% and 95%, respectively.

### 3.3 Ablation and comparison experiments

This section validates the models and methods selected and designed in this study through ablation experiments and comparison experiments. First, a set of comparison experiments was designed to verify the validity of the baseline model selected in this study. Then, a group of ablation experiments based on the modified baseline model was carried out to demonstrate the effectiveness of the improved method adopted in this study. Next, the superiority of the proposed method was verified by designing a set of comparative experiments of multimodal image target detection using different fusion methods and approaches. Finally, a set of ablation experiments was designed to verify the effectiveness of the migration learning and scale -matching methods.

#### 3.3.1 Validation of the baseline framework

In this experiment set, 200 color RGB tea shoot images in Datatset3 were used as the experimental dataset, and it was divided into a training set, a validation set, and a test set at the ratio of 8:1:1. The dataset was trained and validated on models of YOLOv3 (Redmon and Farhadi, 2018), YOLOv4 (Bochkovskiy et al., 2020), YOLOv5, YOLOv6 (Li C. et al., 2022), YOLOv7 (Wang et al., 2022), and YOLOv8 (Jocher et al., 2023), and the test results and model performance are shown in **Table 4**. To ensure fairness, no pre-training weights were used for all models in the training process, and the testing environment and configuration were identical during the experiments.

**Table 4:** Comparative results of detection capabilities of different YOLO frameworks and baseline models.

Method	Parameters	GFLOPs	P	R	mAP50	mAP95
YOLOv3	8666692	12.9	0.686	0.585	0.647	0.254
YOLOv4	5874116	20.0	0.636	0.608	0.799	0.377
YOLOv5	7012822	15.8	0.825	0.733	0.801	0.425
YOLOv6	18500000	45.17	0.779	0.715	0.623	0.322
YOLOv7	6007596	13.0	0.814	0.733	0.703	0.326
YOLOv8	11125971	28.4	0.819	0.732	0.802	0.459

Although YOLOv8 obtained the highest mAP50 value, its number of parameters was 1.5 times larger than that of the YOLOv5s model, and its GFLOPs was 1.8 times higher than that of the YOLOv5s model. YOLOv3, YOLOv4, and YOLOv7, although their number of parameters and GFLOPs were smaller, had relatively low mAP50 values, and especially, YOLOv3 and YOLOv4 had a lower recall. YOLOv6 performed relatively poorly on small targets with dense tea shoots. Overall, YOLOV5 is much smaller and more lightweight than the other models in terms of parameter size and GFLOPS, although its mAP50 value is lower than the highest value. Therefore, YOLOv5s is easier to deploy in practical application scenarios. The above results validate the selection of YOLOv5s as the baseline model in this study.

#### 3.3.2 Validation of baseline model improvements

In this set of experiments, 200 color RGB tea shoot images in Datatset3 were used as the experimental dataset, and they were divided into a training set, a validation set, and a test set at the ratio of 8:1:1. “From Focus to conv” (NoFocus),” From 3693 to 8833” (BH),” From CSP2 to C3_DSConv” (C3_DSConv), and “From PANet to FPN” (FPN) modular architectures and methods were added to the baseline model, respectively. **Table 5** presents the experimental results. Note that no pre-training weights were used for all models during training, and the testing environment and configuration were identical during the experiments.

**Table 5:** Results of ablation experiments with improved baseline model.

NoFocus	BH	FPN	C3_DSConv	Parameters	GFLOPs	P	R	mAP50	mAP95
				7012822	15.8	0.825	0.733	0.801	0.425
√				7012822	15.8	0.835	0.742	0.808	0.427
	√			6746326	16.4	0.841	0.750	0.814	0.444
		√		5979478	14.6	0.836	0.750	0.814	0.441
			√	7016278	13.7	0.826	0.749	0.805	0.426
√	√	√	√	**5715670**	**13.5**	**0.841**	**0.751**	**0.818**	**0.448**

Bold indicates the best experimental results.

The "√" symbol indicates the use of the policy, method, or module.

Overall, the mAP50 of the model was improved after the modules and methods described in Section 2.2.1 were added to the baseline model. Particularly, the recall of tea shoots was significantly enhanced when all the improved methods were used, indicating that our proposed method benefits the detection of tea shoots that are prone to miss-detection. Meanwhile, the number of model parameters and GFLOPs was optimized, which is consistent with our original intention to achieve real-time detection of dense and tiny tea shoots through a lightweight model. Note that the accuracy was significantly improved when the BH strategy was used (aggravating the computation of the early stages of the network). Still, the GFLOPs were also increased by introducing more computation. For this purpose, this study used C3_DSConv to reduce the computational effort, and it can be seen that the GFLOPs were significantly reduced without affecting the accuracy.

Additionally, this study demonstrates the performance of the YOLOv5s model under other BH strategies. The details are presented in **Table 6**. First, it can be seen that relative to the distribution of CSP1_n modules of the original YOLOv5s model, the model detection accuracy and especially the recall were significantly improved by using the method of early calculation of the weighted network. Second, the optimal performance was achieved when the number of CSP1_n modules in the four stages of the backbone was set to 8, 8, 3, and 3, respectively.

**Table 6:** Performance demonstration of the YOLOv5s model under other BH strategies.

Number of CSP1_n modules	Parameters	P	R	mAP50	mAP95
3,6,9,3	7012822	0.825	0.733	0.801	0.425
5,7,6,1	6859030	0.842	0.753	0.811	0.435
4,8,6,2	6889814	0.838	0.740	0.803	0.427
7,8,3,1	6736022	0.844	0.745	0.813	0.442
6,7,4,2	6694934	0.836	0.747	0.813	0.436
10,6,4,1	6705238	0.843	0.746	0.810	0.439
9,8,2,2	6746326	0.839	0.746	0.813	0.443
8,7,3,3	6705238	0.840	0.752	0.811	0.437
9,6,3,3	6705238	0.837	0.747	0.807	0.437
9,9,3,1	6746326	0.839	0.753	0.811	0.441
8,9,2,2	6746326	0.836	0.748	0.811	0.440
8,8,3,3	**6746326**	**0.841**	**0.750**	**0.814**	**0.444**

Bold indicates the best experimental results.

#### 3.3.3 Comparison of multimodal image fusion methods

In this set of experiments, the Dataset3 dataset was used as the experimental dataset, and it was divided into a training set, a validation set, and a test set at the ratio of 8:1:1. However, it is worth noting that the data were preprocessed differently according to different modal fusion methods. This is shown in detail in Section 2.2.2. Also, to further validate the effectiveness and superiority of our proposed baseline model and the multimodal feature fusion model, different experimental models were compared. The performance of the data layer fusion approach was compared on the YOLOv5s baseline and improved models. The performance of the feature layer fusion approach was compared on the CFT model proposed by (Qingyun et al., 2021), the HINet proposed by (Park, 2022), and the YOLOv5-Multimodal model designed in this study. Besides, to show the impact of the baseline improvement-based approach and the introduction of the FFA model, Without_FFA and Without_Improve were added as the ablation experiments for the YOLOv5-Multimodal model. No pre-training weights were used for all models in the training process, and the test environments and configurations were identical during the experiments. **Table 7** presents the specific comparison results.

**Table 7:** Comparison of experimental results of different fusion methods and different models.

Fusion Method	Model	Parameters	P	R	mAP50	mAP95
Data_Fusion1	YOLOv5s_3ch	7012822	0.833	0.740	0.804	0.446
	YOLOv5s_improve_3ch	5715670	0.848	0.754	0.820	0.446
Data_Fusion2	YOLOv5s_4ch	7013974	0.832	0.742	0.808	0.436
	YOLOv5s_improve_4ch	5722230	0.848	0.756	0.824	0.460
Feature_Fusion	GPT(s)	44500982	0.840	0.745	0.810	0.426
	HINet(s)	23738982	0.821	0.731	0.794	0.413
	Without_FFA	11261174	0.807	0.718	0.774	0.394
	Without_Improve	26424892	0.834	0.742	0.809	0.429
	YOLOv5s_Multimodal	**24764092**	**0.850**	**0.759**	**0.827**	**0.447**

Bold indicates the best experimental results.

Overall, the detection accuracy of tea shoots was improved after the multimodal fusion method was used, indicating that the information in different modalities is complementary, and our conjecture in Section 2.2.2 is validated. Regarding the various fusion methods, the multimodal image fusion method using channel-based (Data_Fusion1) achieves a more considerable accuracy gain than the multimodal image fusion method using pixel-by-pixel (Data_Fusion2). However, it increases the number of parameters by a smaller amount. Meanwhile, the multimodal image fusion method with a feature layer introduces more parameters than the multimodal image fusion method based on the data layer. Notably, the mAP50 value of the model decreased when HINet was used directly. Since the HINet model extracts high-frequency information in the frequency domain, so it loses more low-frequency information to guide the detection of small targets. Also, the information is not filtered and aligned in the cross-modal fusion process, thereby introducing some noise that affects the training and convergence of the model. For the GPT model, although the detection accuracy was improved, the use of the multi-head self-attentive mechanism (MHSA) (Vaswani et al., 2017) in the cross-modal fusion module introduces a large number of parameters and computational effort, which is not acceptable in a low-cost agricultural application environment.

**Figure 7:**
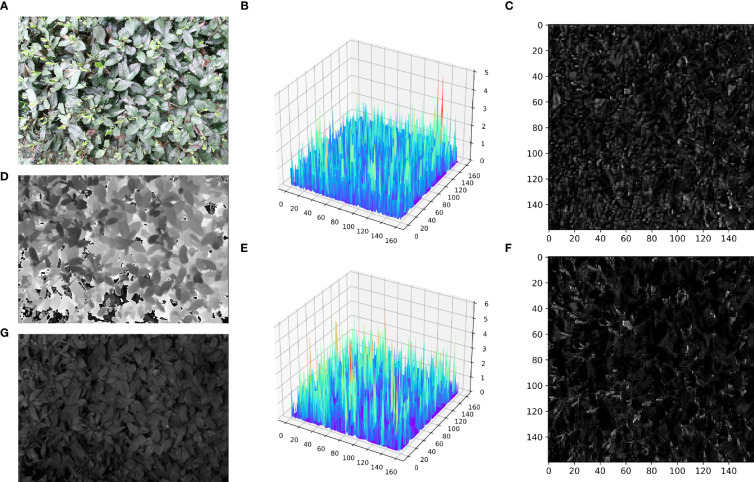
First fusion stage feature map visualization. **(A)** input RGB image; **(D)** input Depth image; **(G)** input IR image; **(C)** feature map of the first fusion stage in the Without_FFA model; **(B)** 3D surface map corresponding to the feature map of the first fusion stage in the Without_FFA model; **(F)** feature map of the first fusion stage in the YOLOv5s_Multimodal model phase in the YOLOv5s_Multimodal model; **(E)** 3D surface map corresponding to the feature map of the first fusion phase in the YOLOv5s_Multimodal model.

In contrast, the model YOLOv5s_Multimodal proposed in this study significantly reduced the number of parameters by purifying, fusing, and enhancing multimodal information in the frequency domain and obtained the best mAP50 value for the tea shoot detection. Meanwhile, by comparing the use of YOLOv5s and YOLOv5s_improve models in different fusion methods, it was found that both YOLOv5s_improve models performed optimally, which again demonstrated the superiority and robustness of the dense and tiny tea shoot detector designed in this study. Note that when the Without_FFA model was used, i.e., directly summing and fusing the features under two modes, the mAP50 value reached the lowest value, which was even lower than that of the unimodal target based on the YOLOv5s model. To analyze this result, the feature maps and 3D surface maps of the first fusion stage of the Without_FFA model and YOLOv5s_Multimodal model are shown in **Figure 7**.

**Figures 7C, F** reveals that when the features extracted in different modalities are directly summed and fused, the resulting feature maps are relatively noisy, and the target edges will be more obvious for the pairs. This is because the coarse and cluttered feature information deteriorates the training and convergence of the model. However, when the FFA module was used to calibrate, purify, and enhance the feature information within and between each modality, the noise in the feature maps was significantly reduced. The tea shoot targets were more prominent, and the edges were more clearly defined. It can be seen from **Figures 7B, E** that in the 3D image with preserved spatial information, the tea shoots do not show significant gradient differences from the background compared to the direct summation mode of the multimodal feature information. However, after the FFA module was used again, the tea shoots exhibited noticeable gradient differences with the background leaves, which is beneficial for identifying and localizing tea shoots. Also, this demonstrates the effectiveness and superiority of our proposed FFA module on the multimodal tea shoot dataset.

#### 3.3.4 Verification of scale matching

**Figure 8:**
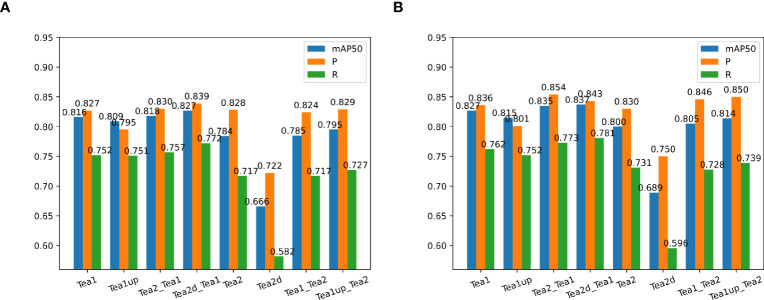
Comparison of experimental results of different models using objective-based scale matching and migration learning. **(A)** indicates the performance on the YOLOv5s model using different scale datasets and training strategies; **(B)** shows the performance on the YOLOv5s_improve model using different scale datasets and training strategies.

To investigate and validate the effectiveness of the scale-matching-based transfer learning method in tea shoot detection, a set of ablation comparison experiments was designed in this study. In the experiments, the color RGB image datasets in Dataset1 and Dataset2 were used as the experimental datasets, called Tea1 and Tea2, respectively, and they were divided into a training set, a validation set, and a test set at a ratio of 8:1:1, and YOLOv5s and YOLOv5s_improve were used as the experimental models. Firstly, this study compared the performance of the two models on Tea1 and Tea2. Secondly, Tea1 was aligned to the scale of Tea2 according to the scale matching method to obtain Tea1up, and the performance of the two models on Tea1up was compared. Similarly, Tea2 was aligned to the scale of Tea1 according to the scale-matching method to obtain Tea2d, and the performance of the two models on Tea2d was compared. Finally, the best weights obtained by training Tea2 and Tea2d were used as pre-training weights to train the model on Tea1 (denoted as Tea2_Tea1 and Tea2d_Tea1, respectively), and the best weights obtained by training on Tea1 and Tea1up were used as pre-training weights to train the model on Tea2 (denoted as Tea1_Tea2 and Tea1up_Tea2). The specific comparison results are given in **Figure 8**. Note that the test environment and configuration during the experiments are identical.

**Figure 9:**
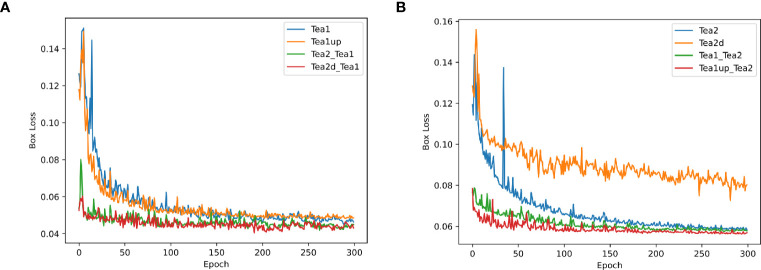
Plots of box loss curves on the YOLOv5s_improve model for different scale datasets and pre-training strategies. **(A)** Box loss profile plots of Tea1 at different scales and pre-training strategies; **(B)** Box loss profile plots of Tea2 at different scales and pre-training strategies.

**Figure 8** shows that the precision, recall, and mAP50 values of the Tea1 and Tea2 datasets were reduced when their scales were aligned to that of the original dataset. This may be because the difficulty of small object detection was exacerbated by the reduced scale of Tea2. Besides, since Tea1 ignored the small object objects in the image edges when increasing the scale, it resulted in fewer small target samples, thus affecting the training and convergence of the model. However, the model accuracy improvement could be stronger when Tea1 and Tea2 were used to guide each other’s learning, and the scale mismatch problem may arise. When the scale-aligned datasets Tea2d and Tea1up were used to guide the model to learn on the Tea1 and Tea2 datasets, respectively, the detection accuracy was significantly improved. Additionally, to more clearly compare the performance of different scale datasets and pre-training strategies during model training and validation, the localization loss curve of the YOLOv5s_improve model on the validation set is shown in **Figure 9**.

**Figures 9A, B** show that when the pre-training weights were used, the initial values of the localization loss were significantly lower, with relatively small curve oscillations, and the loss converged relatively quickly. However, the localization loss converged best when the corresponding scale was used as the pre-training dataset. This also demonstrates the effectiveness of the target-based scale-matching method used in this study in guiding the small target detection task.

### 3.4 Heat map visualization

**Figure 10:**
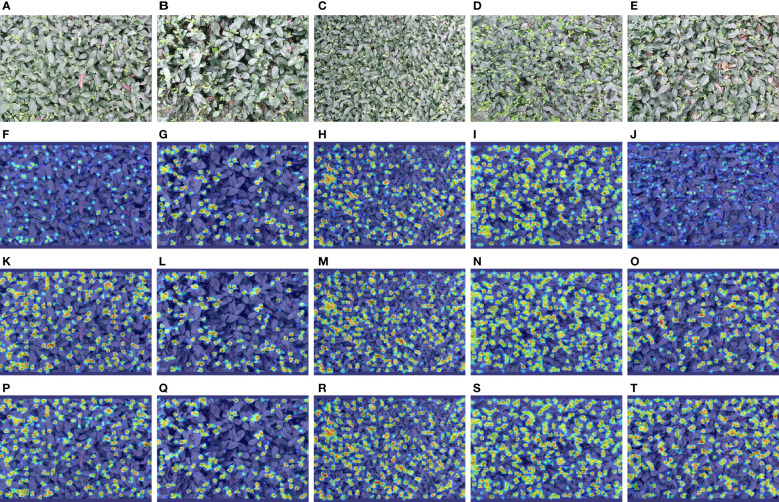
Heat map visualization results for different models with different detection conditions. **(A–E)** The input images; **(F–J)** The results of YOLOv5s; **(K–O)** The results of YOLOv5s_improve; **(P–T)** The results of YOLOv5s-Multimodal.

To more intuitively illustrate the impact of model improvements, explicitly modifying the baseline model for dense and small targets, and the effectiveness of multimodal feature fusion methods, this study used a gradient-weighted class activation mapping (Grad-CAM) (Selvaraju et al., 2016) to visualize the model considering the target based on tea shoots. Grad-CAM can exploit the gradient of any target concept to flow into the final convolution layer, thereby generating a rough localization map and displaying it in the form of weights, where the weight values are shown in red, yellow, green, and blue colors in decreasing order. The redder the color in the corresponding graph, the more critical the region for tea shoot detection. **Figure 10** shows the heat map visualization results for different models under different inspection conditions.

Both YOLOv5s_improve and YOLOv5s-Multimodal models perform better than YOLOv5s in various cases, e.g., the color of tea shoots is similar to the background, tea shoots are relatively sparse, the target scale is rather large, tea shoots are dense and tiny, the color of tea shoots differs from the background, and the target scale is relatively large. Note that when the tea shoot has a similar color to the leaf and its background is difficult, the YOLOv5s model collects minimal information and does not focus on many tiny tea shoot objects. However, YOLOv5s_improve focuses on more tiny tea shoot objects by enhancing the retention and extraction of detailed texture features. However, it is difficult for YOLOv5s and YOLOv5_improve to focus on the groups of tea shoots with high overlap, especially the tiny tea shoots in the overlap case where the tea shoots are relatively dense and overlapping occlusion occurs. However, the multimodal model YOLOv5s-Multimodal has multi-class information input, so it can find more tea shoots and has better segmentation ability for tea shoot groups with high overlap. Besides, it is no longer limited to the part of the stem tip. The model also considers the related connecting stems, leaves, and stems. This demonstrates the superiority of YOLOv5s-Multimodal for tea shoot detection.

### 3.5 Visualization of results

**Figure 11:**
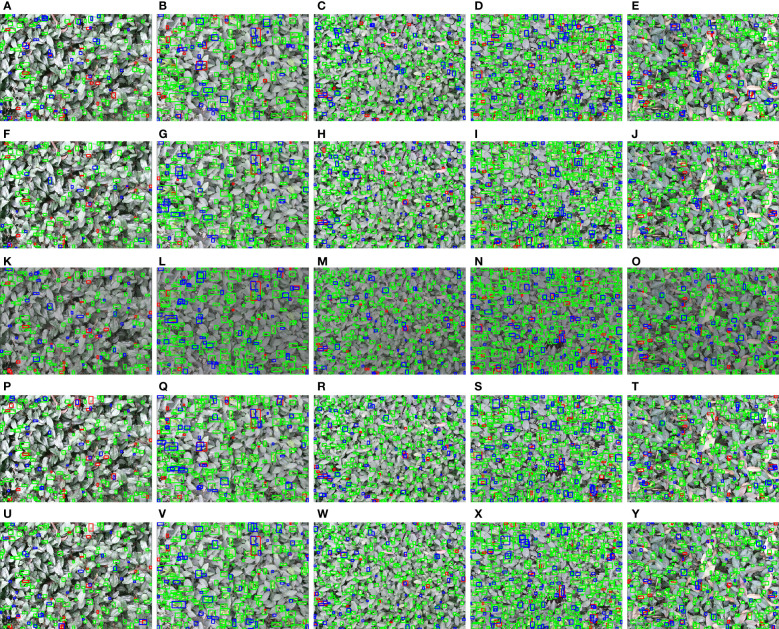
Visualization results of different detection models and methods under different detection conditions. **(A–E)** The test results of YOLOv5s; **(F–J)** The test results of YOLOv5s_improve; **(K–O)** The test results of YOLOv5s_improve_3ch; **(P–T)** The test results of YOLOv5s_improve_4ch; **(U–Y)** The test results of YOLOv5s-Multimodal. The green, blue, and red boxes indicate true positive (TP), false positive (FP), and false negative (FN) predictions, respectively.

To more intuitively compare the performance of different detection models and different fusion methods on the tea shoot detection task in a natural environment, this study performed a comparative analysis of the visualization results of different types of samples after recognition. In this study, YOLOv5s (single modal), YOLOv5s_improve (single modal), YOLOv5s_improve_3ch (multimodal), YOLOv5s_improve_4ch (multimodal), and YOLOv5s-Multimodal (multimodal) were used on the test set of the corresponding experimental dataset. The inference was conducted, and the performance of these models under different detection conditions is shown in **Figure 11**.

In **Figure 11**, the first column shows relatively sparse and tiny tea shoot targets. The second column shows rather large and sparse tea shoot objects. The third column shows relatively dense and small tea-shoot objects. The fourth column shows relatively large and thick tea shoot objects, and the fifth column shows rather complex tea shoot backgrounds. Overall, under different challenging conditions, YOLOv5s_imporve and multimodal-based fusion methods can substantially reduce false negatives (FN), and there is a significant increase in true positives (TP) of YOLOv5s-Multimodal visualization results, which again demonstrates the superiority and robustness of our proposed method.

## 4 Conclusion

This study aims to improve the detection accuracy of dense and tiny tea shoots in a natural environment and realize real-time object detection. In this paper, a real-time dense and small tea shoot target detection algorithm is designed based on multimodal image data, baseline detection model architecture, multimodal image fusion method, scale matching, and migration learning techniques.

First, to make up for dense and tiny tea shoot detection in a complex environment, this paper uses the Conv layer to replace the Focus layer in the YOLOv5s baseline, which is easy to lose detailed information. This helps to extract features for tea shoot detection by enhancing the computation of the early stage of the network while using DSConv to balance the introduced computation and improve the model’s attention to detail texture, and the recall of targets at different scales is enhanced by the FPN structure. The improved model achieves an accuracy of 84.1%, a recall of 75.1%, and a mAP50 value of 81.8% on low-resolution RGB tea shoot images, showing an improvement of 1.6%, 1.8%, and 1.7% compared to the original YOLOv5s model.

Second, to make up for the deficiency of RGB image-based tea shoot detection, two data layer-based multimodal fusion method and one feature layer-based multimodal fusion method are investigated in this paper. Compared with the images based on a single modality, the mAP50 values of Data_Fusion1 and Data_Fusion2 are improved by 1.9% and 2.3%, respectively. Besides, the Feature_Fusion method proposed in this paper achieves the highest mAP50 value of 82.7% at a relatively small number of parameters compared to other feature layer-based multimodal fusion methods. This study mainly introduces a frequency domain-based cross-modal attention fusion module to perform purify, align, fuse, and enhance multimodal information with minor computational effort and parameters. Thus, more complementary information beneficial to detecting dense and tiny tea shoots in complex environments is obtained. Although the feature layer-based multimodal fusion approach proposed in this study introduces a larger number of parameters compared with the data layer-based multimodal fusion approach, the former achieves optimal performance, providing a reference for feature layer-based multimodal fusion approaches. In the future, we will continue to consider the feature layer-based multimodal fusion approach in model lightweight.

Finally, to investigate the differences and effects of training at different scales, this study designed comparison experiments on two tea shoot datasets with target scale differences, and their detection results in different periods were compared. It can be found that small-scale target detection is very complex. To improve the accuracy and recall of tea shoot detection in various scales, this study uses migration learning techniques and scale matching to align datasets of different scales and mutually guide the models to learn at the corresponding scales, thereby improving the performance of small target detection.

However, there are still some drawbacks and limitations in this study. First, although the tea shoot samples used for training in this study are about 50,000, the model’s generalization still needs to be enhanced because the image data are relatively small and do not contain all natural scenes. Secondly, affected by the data acquisition equipment, there are some voids and noises in the acquired depth maps and infrared images, and in the future, we will consider using techniques such as depth estimation, depth enhancement, and image denoising to obtain high-quality depth images and infrared images. Finally, also affected by the data acquisition equipment, the Kinectv2 device could initially acquire high-resolution RGB images; however, since the color camera has a different field of view from the depth camera, the acquired high-resolution images are not aligned with the depth images and infrared images, and the existing alignment techniques based on traditional image processing have some errors. This cannot be neglected in the detection task of dense and small tea shoots. In the future, we will consider introducing a deep learning-based image alignment method and combining it with super-resolution techniques to further improve the detection performance of dense and tiny tea shoots.

## Data availability statement

The datasets presented in this study can be found in online repositories. The names of the repository/repositories and accession number(s) can be found in the article/Supplementary Material.

## Author contributions

LS conceptualized this study, conducted experiments, wrote the original draft, and revised the manuscript. LS and ZL wrote the manuscript and performed the experiments. LS, ZC, and ZL made the experimental plan, supervised the work, and revised the manuscript. BZ and HL performed the data analysis and revised the manuscript. LS and ZC made the experimental plan and revised the manuscript. JM and YW evaluated the developed technique and revised the manuscript. LS designed the experimental plan, supervised the work, and revised the manuscript. All authors have read and agreed to the published version of the manuscript.
